# Titanium metal–organic frameworks for photocatalytic CO_2_ conversion through a cycloaddition reaction†

**DOI:** 10.1039/d4na00535j

**Published:** 2024-08-16

**Authors:** James Kegere, Shaikha S. Alneyadi, Alejandro Perez Paz, Lamia A. Siddig, Afra Alblooshi, Mohamed A. Alnaqbi, Ahmed Alzamly, Yaser E. Greish

**Affiliations:** a Department of Chemistry, College of Science UAE; b Zayed Centre for Health Sciences, United Arab Emirates University Al Ain 15551 UAE y.afifi@uaeu.ac.ae ahmed.alzamly@gmail.com

## Abstract

The elevated levels of CO_2_ in the atmosphere have been a major concern for environmental scientists. Capturing CO_2_ gas and its subsequent conversion to useful organic compounds is one of the avenues that have been extensively studied in the last decade. The photocatalytic cycloaddition of CO_2_ is a promising approach for effective CO_2_ capture and the production of value-added chemicals such as cyclic carbonates. MOF-901, a titanium-based metal–organic framework with hexagonal layers and imine linkages, was successfully oxidized in this study to MOF-997, incorporating amide linkages using Oxone. Both MOFs displayed remarkable photocatalytic activity in CO_2_ cycloaddition under mild conditions, including moderate temperatures and visible light exposure. Particularly noteworthy is MOF-997, exhibiting superior performance with donor–acceptor active sites, achieving a 99.9% yield in catalyzing CO_2_ conversion from styrene epoxide to styrene carbonate under solvent conditions.

## Abstract 2

A schematic representation of the MOF-901 framework, with its imine building units transformed into amide linkages of MOF-997.

## Graphical abstract

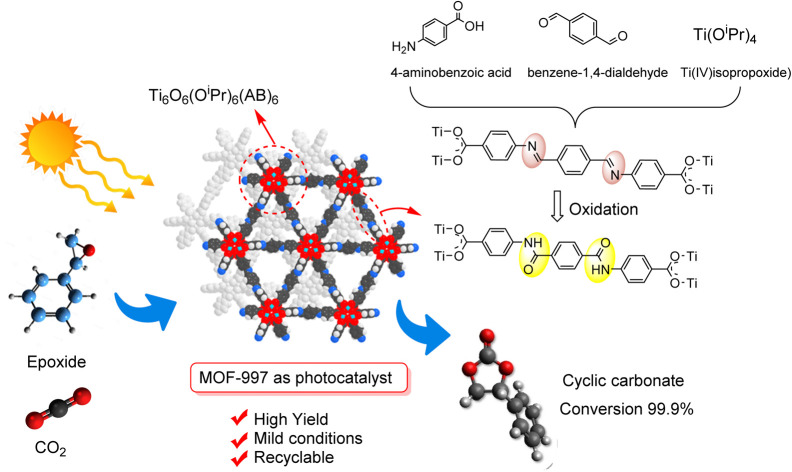

## Introduction

The rising CO_2_ levels are a significant concern that the world is currently facing. The sequestration and conversion of CO_2_ into useful compounds have been receiving much attention as a viable approach to curbing emitted CO_2_ levels that significantly contribute to the greenhouse effect.^1–4^ In particular, the fixation of CO_2_ through cycloaddition to epoxides is a lucrative method for mitigating CO_2_ emissions and producing valuable chemical feedstock.^5^ Metal–organic frameworks (MOFs) have emerged as promising catalysts for photocatalyzing CO_2_ conversion^6–9^ and fixation,^5^ possessing unique features that include a large surface area, tunable pore structure, and low bandgap energy, ensuring light absorption in the visible spectrum.^10–13^ These properties make MOFs particularly attractive for use in photocatalysis. Moreover, the presence of multivalent metal clusters and active sites in MOFs increases the likelihood of the existence of Lewis acid and Brønsted character, which, in synergy, promote CO_2_ fixation *via* cycloaddition under mild conditions.^14^

Cycloadditions play an important role in the synthesis of organic compounds used in various industries, including medicine and chemical engineering.^14–17^ To catalyze this promising and atom-economical reaction, many heterogeneous catalysts have been developed, including zeolites, polymers, porous carbon, ion-liquid supported solids and MOFs.^17^ The main drawback, however, has been the reliance on harsh solvents and elevated temperature and pressure. Consequently, the focus of research has shifted towards developing new heterogeneous catalysts capable of performing photocatalytic cycloaddition reactions under mild and practical conditions, such as room temperature and atmospheric pressure.^17^

Titanium-based MOFs present intriguing potential for various photocatalytic applications, yet their utilization in photocatalytic CO_2_ cycloaddition reactions is scarcely reported.^10^ This contribution explores MOF-901,^18^ a titanium MOF exhibiting a hexagonal layer (hxl) structure, to study its photocatalytic properties in CO_2_ cycloaddition reactions. The imine linkages within MOF-901 can be transformed into amide linkages, with the hypothesis that the oxidized MOF would demonstrate enhanced photocatalytic activity due to its interaction with CO_2_*via* donor–acceptor sites (amide–CO_2_). Experimental oxidation of imine linkages^19^ in MOF-901 to amide bonds in MOF-997 is achieved using Oxone-based oxidation conditions.^19^ The conversion of imine to amide units in MOF-997 enhances the photocatalytic properties of the resulting MOF-997, surpassing the pristine MOF-901 in converting CO_2_ and styrene oxide into styrene carbonate with a quantitative yield. Notably, MOF-997 demonstrates superior performance compared to other materials used in this photocatalytic application^15–17,20^ and facilitates the cycloaddition of CO_2_ under practical and mild conditions. Our findings demonstrate that MOF-997 outperforms MOF-901 and many other catalysts^14–17^ in solvent-based and solvent-free cycloaddition reactions, offering a high degree of recyclability.

## Methods

The structure and crystallinity of MOF-901 and MOF-997 were assessed through powder X-ray diffraction (PXRD). Fourier-transform infrared (FTIR) and solid-state ^13^C multiple cross polarization magic-angle spinning nuclear magnetic resonance (^13^C-CP-MAS NMR) spectroscopy confirmed the imine-to-amide transformation. A scanning electron microscope (SEM) was employed to analyze the MOF morphology, particularly focusing on the effects of oxidation on MOF-901. Porosity was estimated using N_2_ sorption analysis at 77 K, while UV-Vis diffuse reflectance spectroscopy (UV-Vis DRS) was used to determine the bandgap energy. Thermal stability was investigated through thermogravimetric analysis (TGA). Photocatalytic activity was assessed by studying the cycloaddition of CO_2_ and phenyl epoxide to form styrene carbonate. Density functional theory (DFT) calculations were conducted by the projector augmented wave (PAW) method as implemented in the GPAW code.^21,22^ The electronic orbitals were expanded using a linear combination of atomic orbitals (LCAOs) with double-zeta-polarized (dzp) quality. The reciprocal space was sampled at the *Γ* point only due to the large size of the unit cell (side > 23 Å along the periodic direction). The PBE^23,24^ exchange correlation functional and the Grimme's^23^ D3 parametrization of the van der Waals corrections were used. Geometry optimization was deemed converged when the atomic forces fell below 0.01 eV Å^−1^. Our relaxed bond lengths deviate from the model structure by less than 0.15 Å.

## Results and discussion

**Fig. 1:**
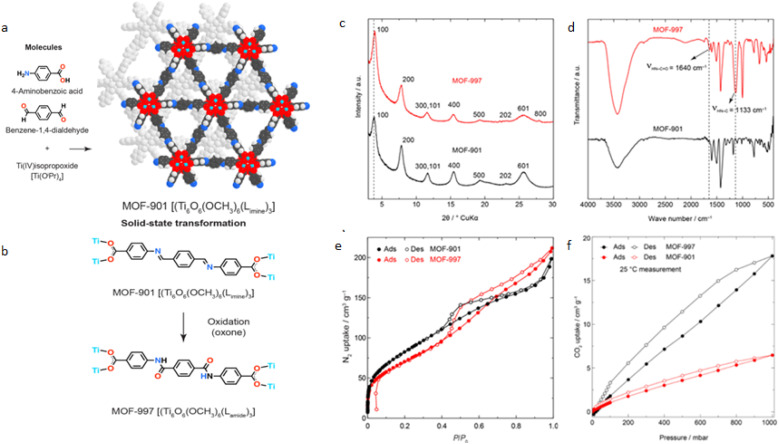
Synthesis and characterization of MOF-901 and MOF-997. (a) Structure of MOF-901, (b) chemical transformation of imine in MOF-901 to amide in MOF-997, (c) PXRD patterns of MOF-997 slightly shifted to the right compared to MOF-901; (d) overlay of the FTIR spectra of MOF-901 and MOF-997, showing the appearance of amide vibrations in MOF-997; (e) N_2_ sorption isotherms of MOF-901 and MOF-997; the porosity of MOF-997 reduced after oxidation due to the mass increase of amide units; (f) overlay of the CO_2_ sorption isotherms of MOF-901 and MOF-997. The hysteresis of MOF-997 isotherms indicating a strong interaction between the MOF and CO_2_.

MOF-901 was synthesized as previously published. The as-synthesized MOF was then solvent-exchanged and activated under dynamic vacuum before undergoing post-synthetic oxidation to yield MOF-997, which has a dark yellow color. An attempt to synthesize the cluster followed by imine conversion *in situ* was not successful. The PXRD pattern of MOF-901 exhibited characteristic peaks at low 2*θ*: 3.75° and 7.67°. In our study, notable shifts and broadening in the X-ray Diffraction (XRD) patterns were observed, which can be attributed to the employment of a solvent system distinct from those reported in a previous study.^18^ The choice of the solvent molecule is known to significantly influence the nucleation and growth processes of MOF structures, thereby affecting their crystal size, morphology, and even the formation of specific phases. In our studies, the solvent system used seems to have induced alterations in the MOF's crystal structure, potentially through the inclusion of solvent molecules within the framework. This resulted in modifications to the lattice parameters, as evidenced by the observed shifts in the XRD peaks. Additionally, the broadening of these peaks suggests variations in crystal size and/or the introduction of structural disorder, possibly due to the differential solvation effects or kinetic factors associated with the chosen solvent system. Such findings underscore the critical role of solvent selection in the synthesis of MOFs, not only for controlling the physical properties of the resulting materials but also for tuning their structural characteristics at the atomic level. The absence of major peaks in the region above 10° indicates the successful transformation of the imine MOF into the amide MOF (Fig. S1†). MOF-997 displayed a similar PXRD pattern to that of MOF-901, indicating that the oxidation reaction of MOF-901 did not disrupt the crystalline structure of MOF-901 (Fig. 1a).

In both MOF-901 and MOF-997 models, each Ti(iv) center exhibits octahedral geometry, but it favors the formation of a distorted square planar pyramidal environment with one axial methoxy anion (CH_3_O^−^) and two shared carboxylates (bidentate k^2^-COO^−^) on the equatorial plane. These groups are linked *via* the oxygen atom, resulting in Ti–O bond distances of 2.0 and 1.95 Å, respectively. Additionally, there are three μ^3^-oxo groups (one in the axial position and two on the equatorial plane) shared between three adjacent Ti atoms with Ti–O bonds ranging from 1.96–1.98 Å and a Ti–O–Ti bond angle of 135°. The extended structure of the MOF is generated by linking adjacent hexameric Ti clusters with imine-based (or amide-based in the oxidized variant) linkers. Staggered layers in the MOF create hexagonal pores with a diameter of 11 Å. Notably, oxidizing imine to amide units does not significantly alter the pore size of the oxidized MOF, MOF-997. The imine-to-amide conversion of MOF-901 into MOF-997 was tracked using FTIR spectroscopy. The FTIR spectrum of MOF-901 exhibited characteristic imine linkages with vibrations at 1626 cm^−1^ and 1199 cm^−1^ corresponding to C=N and C–C=N bonds, respectively. In the FTIR spectrum of MOF-997, these vibrations shifted to the range of 1645–1650 cm^−1^ and 1133 cm^−1^, providing clear evidence of amide formation (–HN–OC– and HN–C, respectively), as depicted in Fig. 1b. To assess the porosity of both MOF-901 and MOF-997, N_2_ sorption measurements at 77 K were conducted. MOF-901 showcased a type-II isotherm, yielding a calculated Brunauer–Emmett–Teller (BET) area of 310 m^2^ g^−1^. Similarly, MOF-997 displayed a type-II isotherm with a BET area of 262 m^2^ g^−1^ (Fig. 1c). The decline in the surface area of amide-MOFs can be ascribed to multiple factors. Mainly, the reduction in the gravimetric surface area arises from oxidation. Furthermore, post-synthetic modification contributes to diminished crystallinity, further reducing the surface area. Fig. 1c overlays the N_2_ isotherms of MOF-901 and its oxidized variant. The isotherm of MOF-997 exhibits H_3_ hysteresis, likely linked to decreased crystallinity post-synthetic oxidation and the interaction between the adsorbate and the amide-incorporated framework. The pore diameter estimated from N_2_ isotherms revealed the presence of micropores in both MOFs (Fig. S2†). This characteristic is well-suited for CO_2_ uptake due to the strong interaction between CO_2_ and the MOF framework. MOF-901 adsorbs only 6 cm^3^ g^−1^ of CO_2_ at room temperature and 1 bar, while MOF-997 exhibits significantly higher capacity, reaching 17.5 cm^3^ g^−1^ under the same conditions (Fig. 1d). Additionally, notable variation in the CO_2_ isotherm of MOF-997 indicates a strong interaction between CO_2_ and the amide framework, a feature not observed in MOF-901.

**Fig. 2:**
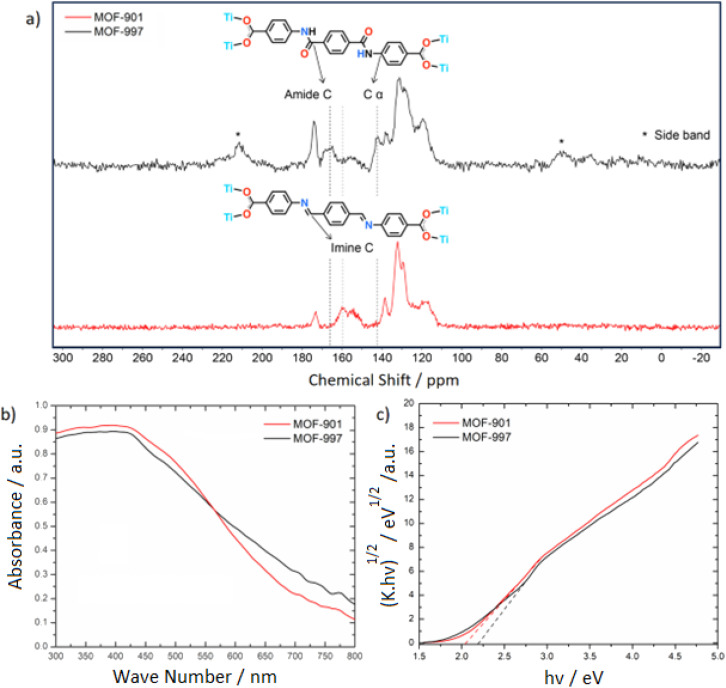
(a) Overlay of the solid-state ^13^C-CP-MAS NMR spectra of MOF-901 and MOF-997, showing the disappearance of imine linkages in MOF-901 and the appearance of amide linkages in MOF-997; (b) bandgap determination of MOF-901 and MOF-997 through the Tauc plot; (c) UV-Vis DRS spectra of MOF-901 and MOF-997. Both MOFs strongly absorb visible light at 450 nm.

The complete oxidation of MOF-901 was further confirmed through ^13^C multi-CP-MAS NMR (Fig. 2a). The imine chemical shift at 159.6 ppm disappeared after the post-synthetic oxidation of MOF-901. Furthermore, the appearance of an amide chemical shift at 166.5 ppm indicated the successful conversion of imine to amide. MOF-901 demonstrates semiconductive behavior owing to its hexameric titanium units and highly conjugated imine linkers.^18^ The electronic properties and bandgap energy of both MOFs were examined using UV-Vis DRS, as illustrated in Fig. 2b. The calculated bandgap for MOF-901, determined through a Tauc plot, remains consistent at 2.03 eV, aligning with the reported value.^18^ In contrast, MOF-997 showcases a bandgap energy of 2.19 eV (Fig. 2c). This narrow bandgap holds significance in photocatalysis, enabling the MOFs to efficiently absorb visible light.

Following the confirmation of the successful syntheses of MOF-901 and its oxidized variant, an examination of their thermal stability was conducted using TGA (Fig. S3†). The TGA curves exhibit notable stability for both MOFs, with the onset of degradation at 300 °C.

**Fig. 3:**
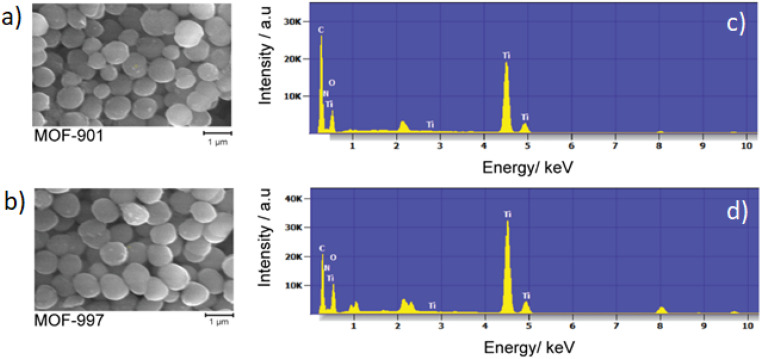
FE-SEM images (a and b) and EDX patterns (c and d) of MOF-901 (a and c) and MOF-997 (b and d).

After confirming the imine-to-amide transformation, the morphology of both MOF-901 and MOF-997 was investigated. Field emission SEM (FE-SEM) analysis at low magnification displayed well-distributed nano-sized particles, averaging 300 nm in size (Fig. 3). Under high magnification, FE-SEM observations revealed smooth surfaces for both MOFs. Notably, there were no observable differences in texture between the particles of MOF-901 and MOF-997, indicating that the conversion from imine to amide did not induce structural alterations in MOF-901. The subsequent analysis using energy dispersive X-ray spectroscopy detected changes in the percentage abundances of various elements. For instance, the percentage of carbon in pristine MOF-901 is higher than in its oxidized counterpart, while the proportion of oxygen increases in the oxidized MOF (Table S1†).

DFT calculations were utilized to compute electronic gaps and confirm the semiconducting properties of the MOFs. The bandgaps for the amide MOF were calculated to be 2.719 and 2.767 eV at the LDA and PBE levels, respectively, closely aligning with our experimental findings. For the imine MOF, the Kohn–Sham gap was reduced to 2.191 and 2.239 eV for LDA and PBE functionals, respectively. This implies that DFT-PBE predicts an increase in the band gap when transitioning from imine to amide functionalization. It should be noted that these values are qualitative, as commonly used DFT-GGA functionals tend to underestimate the optical gaps. More sophisticated methods (such as hybrids or GW0) are needed to reduce differences with experimental results. However, due to the substantial size of our system, these advanced methods are beyond the scope of this study.

**Fig. 4:**
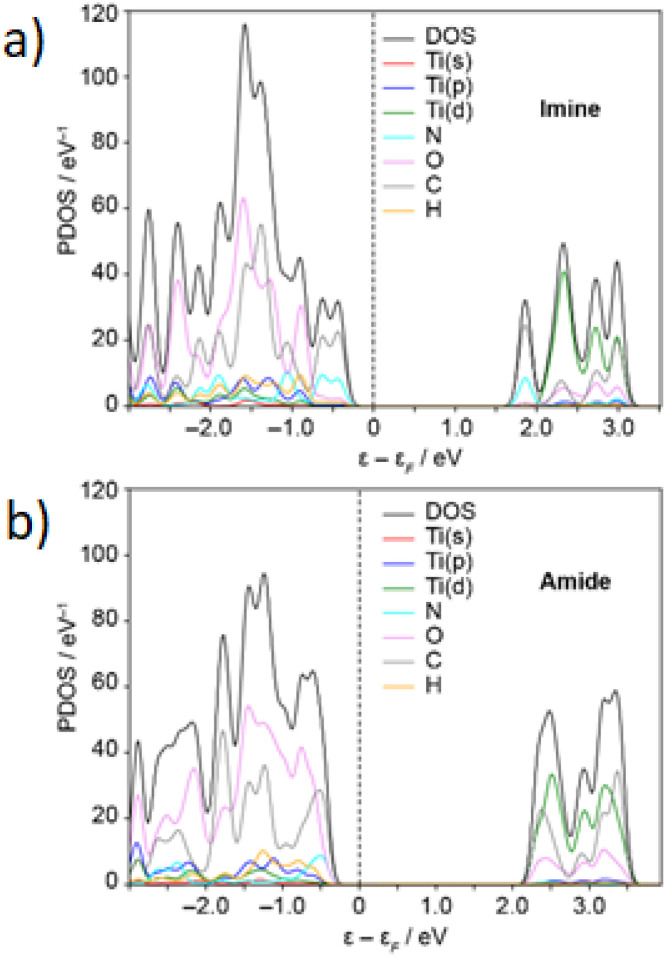
Electronic projected density of states (PDOS, in eV^−1^) for MOF-901 (imine linkages; a) and MOF-997 (amide linkages; b). MOFs computed at the PBE level. Energies (in eV) relative to the Fermi level. The projection of the bands onto the titanium s, p and d atomic orbitals is shown.

**Fig. 5:**
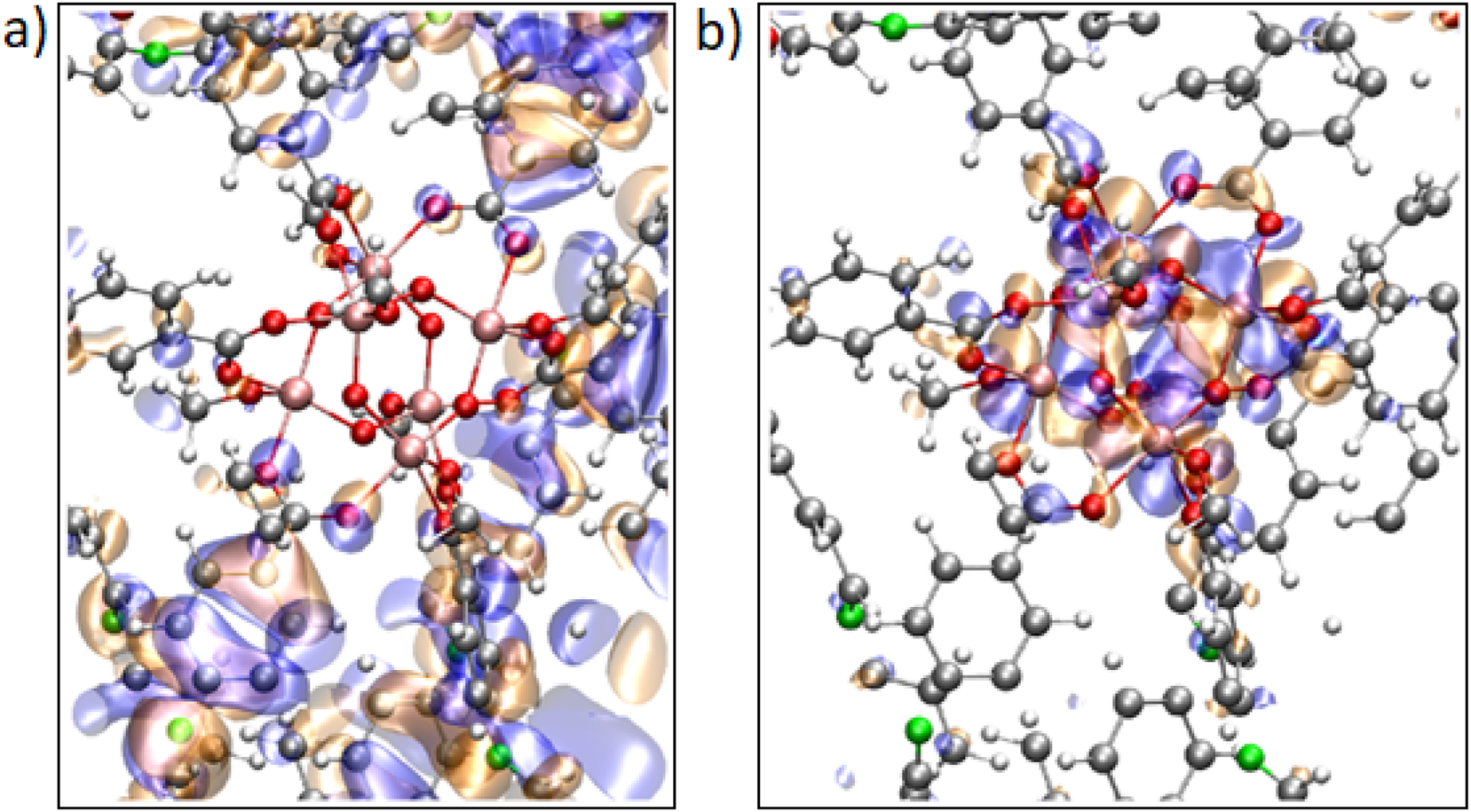
(a) Calculated isosurface representation (isosurface value ±0.1) of the highest occupied band; (b) an unoccupied level with a large component on Ti(3d) atomic orbitals for the imine MOF. The corresponding bands for the amide MOF are similar (not shown). The gray, red, green, pink, and white spheres represent the C, O, N, Ti, and H atoms, respectively. The square planar pyramidal environment around each Ti ion is evident.

The density of states for MOF-901 and MOF-997 is illustrated in Fig. 4, with our analysis commencing through PBE calculations to initially explore the electronic structure of these MOFs. Initially, the average charges of 2.097 and 2.081*e* for the Ti ions in MOF-901 and MOF-997, respectively, were revealed through a Bader charge analysis. Following this, the electronic Projected Density of States (PDOS) around the Fermi level was plotted, confirming the semiconductor characteristics of both MOFs. The highest occupied bands have negligible contributions from the Ti atoms and are primarily influenced by C atoms, with additional contributions from the N and O atoms for the imine and amide MOFs, respectively. At 2.4–2.5 eV above the Fermi level, we observed the emergence of unoccupied bands, with major contributions from Ti-3d and C orbitals for both MOFs, along with minor contributions from N and O atoms for MOF-901 and MOF-997, respectively. This indicates that the UV-Vis absorption involves a ligand-to-metal charge transfer. The findings are further confirmed by an isosurface representation of the band-edge Kohn–Sham orbitals (Fig. 5). In the analysis of both MOF structures, it was observed that the Highest Occupied Molecular Orbital (HOMO) primarily resides within the π-conjugated cores of the benzoate ligands. Conversely, bands approximately 2.2 eV above the Fermi level display a notably stronger component originating from the Ti(3d) orbitals. This observation further substantiates the idea that the oxidation of imine to amide in MOF-901 has notably improved its photocatalytic characteristics.

**Fig. 6:**
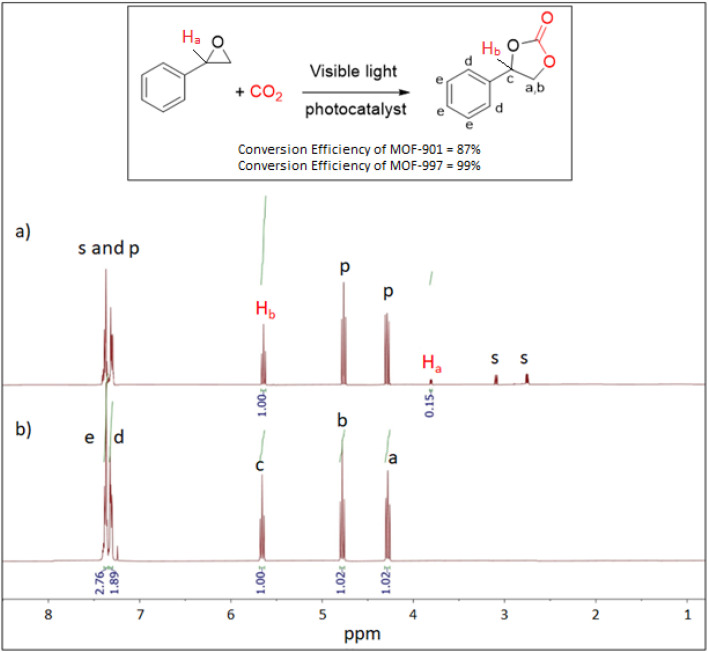
^1^H-NMR spectra of the styrene carbonate product catalyzed by (a) MOF-901, and (b) MOF-997. Inset: a scheme showing the cycloaddition of CO_2_ and styrene oxide to form styrene carbonate.

Our investigation into the electronic structure and band alignment of our MOFs, coupled with CO_2_ isotherms, has indicated that MOF-901 and MOF-997 are well-suited for photocatalyzing the cycloaddition reaction of CO_2_ under visible light. In our initial test, conducted in an acetonitrile and methanol mixture (3 : 1 v/v), MOF-901 yielded 87% conversion (Fig. 6a). As expected, MOF-997, demonstrating superior photocatalytic activity in the CO_2_ and styrene oxide cycloaddition reaction, achieved a nearly quantitative yield (99.9%; Fig. 6b) (Table 1). We conducted control experiments under various conditions to validate the results. Without a co-initiator, the conversion was negligible, irrespective of the experimental conditions, and the same held true for the reactions carried out without MOF catalysts (MOF-901 and MOF-997). Similar low yields, and in some cases zero conversion, were observed in the reactions performed in the dark or solely in the presence of heat. This underscores the irreplaceable role of light in the reaction (Table 2).

**Table 1:** NMR peak assignments for the photocatalytic conversion of styrene oxide to styrene carbonate using MOF-901 and MOF-997 in the presence of CO_2_

Chemical shift (ppm)	Assignment	Multiplicity	MOF-997 (complete reaction)	MOF-901 (incomplete reaction)	Notes
2.71–2.93	Methylene proton (CH_2_) adjacent to oxygen	Multiplet	Not observed	Observed	Absence in MOF-997 suggests full conversion in MOF-997; less in MOF-901
3.83 (H_a_)	Methine proton (CH) adjacent to phenyl	Multiplet	Not observed	Observed	Absence in MOF-997 suggests full conversion in MOF-997; less in MOF-901
4.37–4.72	Methylene protons adjacent to oxygen (CH_2_)	Multiplet	Observed	Observed	Presence in both
5.48 (H_b_)	Methine proton (CH)	Multiplet	Observed	Observed	Higher intensity suggests complete conversion in MOF-997
7.25–7.32	Aromatic protons	Multiplet/doublet	Observed	Observed	Presence in both

**Table 2:** Cycloaddition of CO_2_ and styrene oxide under acetonitrile/methanol solvent conditions

Entry	Photocatalyst^a^	Catalyst (mol%)	Yield^b^ (%)	TON^c^	TOF^d^ (h^−1^)
1	MOF-901, *n*-Bu_4_NBr, light	0.005	87	56	2.3
2	MOF-997, *n*-Bu_4_NBr, light	—	99.9	—	—
3	*n*-Bu_4_NBr, no catalyst, light	—	0	—	—
4	MOF-901 light	0.005	0	0	0
5	MOF-901, *n*-Bu_4_NBr, no light	0.005	0	0	0
6	MOF-901, no light	0.005	0	0	0
7	*n*-Bu_4_NBr, no catalyst, no light	—	6	3.9	0.2
8	MOF-901, *n*-Bu_4_NBr, no light, heat (353 K)	0.005	40	25.8	1.1
9	TiO_2_, *n*-Bu_4_NBr, light	—	4	—	—
10	TiO_2,_ light	—	0	—	—
11	No *n*-Bu_4_NBr, no catalyst, light	—	0	—	—
12	Ti-oxo cluster	—	33	—	—
13	Amide linker	—	45	—	—
14	Imine linker	—	16	—	—

a Reaction conditions: styrene oxide (48.06 mg, 0.44 mmol), photocatalyst (10 mg), *n*-Bu_4_NBr (9 mg, 0.028 mmol), and 0.045 mmol carbon dioxide at 353 K and 24 h in 4 mL of acetonitrile : methanol (3 : 1 v/v).

b Conversion determined by ^1^H NMR.

c TON: turnover number = (mmol of product)/(mmol of catalyst).

d TOF: turnover frequency = (mmol of product)/(mmol of catalyst)(reaction time, hours).

**Fig. 7:**
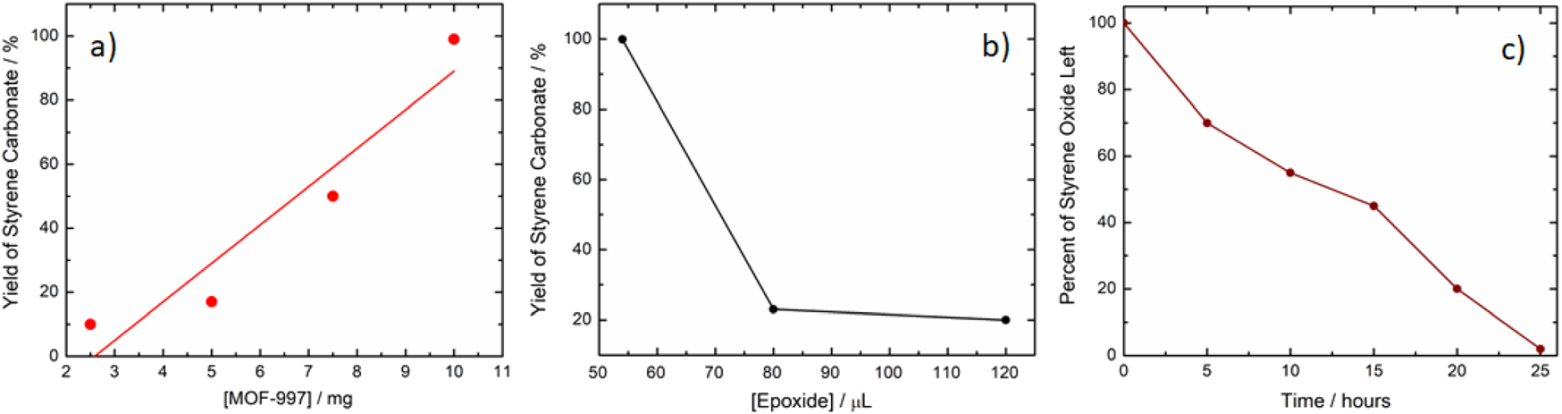
(a) Kinetics study of yield variation with increasing MOF concentration, (b) styrene carbonate yield *versus* styrene oxide concentration for a fixed amount of MOF-997 (10 mg), and (c) kinetics study of epoxide consumption over time.

MOF-997 displayed remarkable stability over three successive cycles without any loss of performance (Fig. S4†). This excellent photocatalytic activity of MOF-997 has prompted us to investigate the mechanism and reaction order of CO_2_ cycloaddition by monitoring the progression of styrene oxide consumption, expressed as the percentage reduction in volume over time. To do so, we conducted experiments involving different concentrations of styrene oxide at a fixed concentration of MOF-997 and TBAB, keeping all other variables constant. We also tested various concentrations of MOF-997 under constant conditions and examined the change in the concentration of styrene oxide over time. The results indicate a first order reaction system with non-first-order kinetics (Fig. 7). This behavior is similar to a reported study,^25^ where ZnBr was used as a co-catalyst for the cyclization reaction of CO_2_ and styrene oxide.

The investigation into the differential performance of MOF-997 and MOF-901 in the CO_2_ cycloaddition reaction yielded intriguing insights. Utilizing Density Functional Theory (DFT) electronic simulations, it was discovered that both MOF-901 and MOF-997 possess susceptible Ti–carboxylate bonds (Fig. 4 and 5). This characteristic allows facile dissociation, enabling the coordination of styrene oxide with active Ti centers, aligning with prior findings on polymerization reaction mechanisms.^26^ Furthermore, an interesting observation surfaced concerning the robust interaction between CO_2_ and amide linkages. This interaction potentially alters CO_2_ geometry and significantly enhances its binding affinity to the styrene oxide intermediate. Supported by a recorded 20.7 kJ mol^−1^ binding energy for CO_2_–amide interaction, surpassing CO_2_–imine interaction by 3.3 kJ mol^−1^,^27^ these CO_2_–amide binding sites, evident in the CO_2_ sorption isotherm (Fig. 1d), play a pivotal role as donor–acceptor units, notably augmenting MOF-997's photocatalytic activity. This distinctive attribute of MOF-997, featuring a diverse array of photocatalytic sites including titanium-oxo clusters and amide units, underscores its marked superiority over MOF-901 in the CO_2_ cycloaddition reaction. The substantiation of this phenomenon was attained through the synthesis and evaluation of imine^28^ and amide^29^ linkers alongside Ti hexameric clusters^30^ in the CO_2_ cycloaddition to styrene oxide (Scheme S1†). Notably, the amide linker outperformed the imine counterpart, showcasing a conversion efficiency of 45% (Fig. S5†) in contrast to 16% for the imine linker (Table S2†). These findings illuminate the critical role of MOF-997's structural attributes in fostering enhanced photocatalytic performance, offering valuable insights for advanced catalytic applications.

Two additional epoxides, 1,2-epoxy-3-phenoxypropane and 2-(4-chloro phenyl)oxirane, were examined employing MOF-901 and MOF-997 photocatalysts (Fig. S6†). MOF-997 demonstrated conversions of 46% and 35% for 1,2-epoxy-3-phenoxypropane and 2-(4-chloro phenyl)oxirane, respectively, into the corresponding carbonate products, outperforming MOF-901, which achieved conversions of 38% and 24%, respectively. The lower yields of carbonates from 1,2-epoxy-3-phenoxypropane and 2-(4-chloro phenyl)oxirane with MOF-901 and MOF-997 photocatalysts can be attributed to factors such as differing bulky functional groups impacting substrate reactivity and access to active sites, potential variations in catalytic activity between the MOFs towards different substrates, varying interactions between epoxides and MOF active sites, potential steric hindrance from bulky groups limiting accessibility, and the electronic effects of the chlorine atom (halogen) in 2-(4-chloro phenyl)oxirane changing its reactivity in the photocatalytic process.

In the pursuit of greener catalytic conditions, the potential of our MOFs to facilitate this reaction without any solvent was explored. Surprisingly, both MOFs exhibited remarkable ability to fully convert CO_2_ and styrene oxide into styrene carbonate (Table S3†).

The conversion of the hexagonal layer titanium metal–organic framework MOF-901 to MOF-997, featuring a shift from imine to amide linkages, stands as a key enhancement in the realm of catalytic CO_2_ conversion. This transformation capitalizes on fundamental structural changes, particularly the increased stability and reactivity conferred by the amide groups, owing to their stronger resonance and hydrogen bonding capabilities. The observed superior catalytic performance of MOF-997 can be attributed to several key mechanistic factors. Firstly, the sensitive electron density and delocalization in amide bonds significantly enhance their ability to interact with and activate CO_2_ molecules, promoting the subsequent catalytic reaction. Moreover, the presence of donor–acceptor sites within MOF-997 plays a crucial role in facilitating efficient electron transfer, key for activating CO_2_ and driving the desired cycloaddition reaction. The structural rigidity imparted by the amide linkages further aids in stabilizing reaction intermediates and optimizing the positioning of catalytic sites, eventually ending in an exceptional catalytic activity observed during CO_2_ conversion. Mechanistically, MOF-997 harnesses visible light irradiation to activate and adsorb CO_2_*via* amide sites, leveraging its electron-rich nature to facilitate electron transfer and subsequent conversion into valuable intermediates. This well-coordinated series of events elucidates the precise role played by the transformation from imine to amide linkages in empowering MOF-997, shedding light on its remarkable ability to activate and catalyze CO_2_ conversion with exceptional efficiency.

**Fig. 8:**
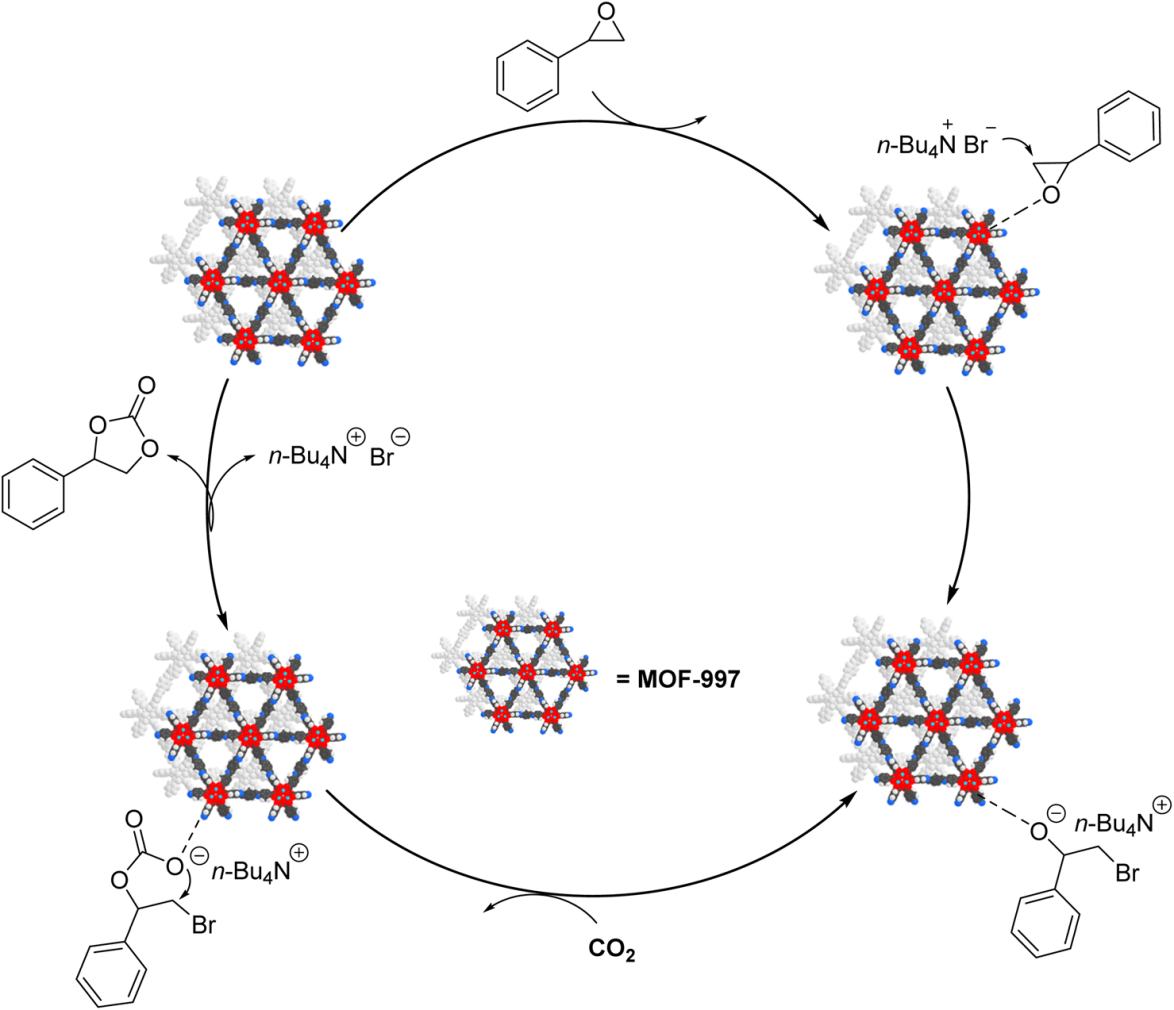
The potential mechanism depicting the cycloaddition reaction between CO_2_ and epoxides facilitated by MOF-997.

The proposed mechanism emphasizes the crucial role of Ti(iv) isopropoxide clusters and amide groups within the MOF structure, acting as Lewis acidic sites that initiate the activation of epoxy rings through coordination with the oxygen atom. This activation facilitates a subsequent nucleophilic attack by Br^−^ ions from tetrabutylammonium bromide (*n*-Bu_4_NBr, also known as TBAB), leading to the opening of the epoxy rings. Subsequently, CO_2_ molecules integrate into the opened epoxy ring and interact with the oxygen anion to produce an alkylcarbonate salt. As the ring closes, cyclic carbonates form, concurrently enabling the regeneration of the MOF-based catalyst (see Fig. 8). This detailed mechanism not only delineates the stepwise progression of the conversion process but also underscores the pivotal role played by MOF-997 and its interaction with TBAB in enabling the formation of valuable cyclic carbonates from epoxides and CO_2_. Furthermore, the catalyst's recyclability further enhances its appeal for sustainable catalytic applications, offering both efficiency and environmental advantages.

The characteristics of MOFs, specifically their pore size and surface area, play a pivotal role in determining the localization of catalytic reactions within their structures. In our study, the differences between MOF-901 and MOF-997, with the respective pore sizes of 14.1 and 14.5 angstroms and surface areas of 310 and 265, offer valuable insights into the probable sites of catalytic activity. MOF-901, with its smaller pore size but higher surface area, suggests a tendency toward surface-based reactions. The correlation observed between the higher surface area and potential surface-driven reactions aligns with established principles, indicating that a more extensive surface area can offer more active sites for catalytic activity. Conversely, MOF-997, characterized by larger pore sizes but a slightly diminished surface area, hints at a different scenario. The larger pores suggest the likelihood of reactions occurring within these spaces. However, the observed lower surface area could imply challenges related to diffusion limitations within these pores. This scenario could potentially hinder access to active sites, resulting in lower yields during the catalytic process. This interesting contrast between MOF-901 and MOF-997 underscores the significance of pore size and surface area in dictating where catalytic reactions predominantly take place within MOF structures. Further investigation and optimization considering these structural attributes could aid in tailoring MOFs for enhanced catalytic performance and yields. The decrease in yields observed when using bulkier substrates, such as larger epoxides, within the MOF catalytic framework can be attributed to multiple factors. These substrates, due to their larger molecular size and increased steric hindrance, face challenges in accessing the active sites within the MOF structure, potentially impeding their interaction and reducing overall efficiency. Diffusion limitations within smaller pores or restricted spaces hinder the substrates' mobility, limiting their access to the catalytic sites and thus reducing reaction rates. Additionally, the reduced surface accessibility of bulkier substrates to deeper reactive sites and potential mismatches in geometry with the catalytic centers can lead to lower affinity and decreased reactivity. Addressing challenges such as steric hindrance, diffusion, surface accessibility, and compatibility between the catalyst and substrate is essential to enhancing yields when dealing with larger, bulkier substrates within MOF catalytic systems.

## Conclusion

The transformation of MOF-901, initially comprising imine units, into MOF-997, now integrating amide linkages, was achieved without compromising the structural integrity of MOF-901. This modification in the MOF backbone resulted in a notable enhancement of its photocatalytic properties, particularly in the conversion of CO_2_ and styrene oxide into styrene carbonate when using an acetonitrile and methanol mixture. Even more remarkable, both MOF-901 and MOF-997 demonstrated exceptional performance in the cycloaddition of CO_2_, achieving quantitative yields under solvent-free conditions. This study introduces two Ti-MOFs, one of which is new, as highly promising photocatalysts. These MOFs are straightforward to synthesize and exhibit thermal and chemical stability. More importantly, our photocatalysts operate under mild conditions, making them suitable for green photochemical applications.

## Conflicts of interest

The authors declare no conflict of interest.

## Supplementary Material

NA-006-D4NA00535J-s001

NA-006-D4NA00535J-s002

NA-006-D4NA00535J-s003

NA-006-D4NA00535J-s004

NA-006-D4NA00535J-s005

NA-006-D4NA00535J-s006

NA-006-D4NA00535J-s007

NA-006-D4NA00535J-s008

NA-006-D4NA00535J-s009

NA-006-D4NA00535J-s010

## Data Availability

The data supporting this article have been included as part of the ESI.†
