# Supplementary figures and images for: Titanium metal–organic frameworks for photocatalytic CO2 conversion through a cycloaddition reaction
Source: Nanoscale Adv. 2024 Aug 16;6(19):4804–13. doi: 10.1039/d4na00535j (PMC11391913; doi:10.1039/d4na00535j)

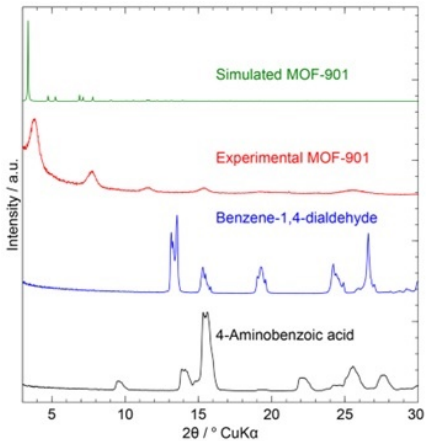

Supplement: NA-006-D4NA00535J-s001 [file NA-006-D4NA00535J-s001.pdf]

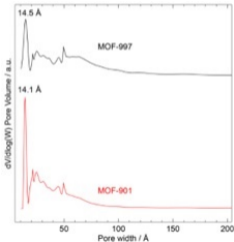

Supplement: NA-006-D4NA00535J-s003 [file NA-006-D4NA00535J-s003.pdf]

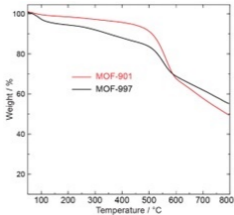

Supplement: NA-006-D4NA00535J-s004 [file NA-006-D4NA00535J-s004.pdf]

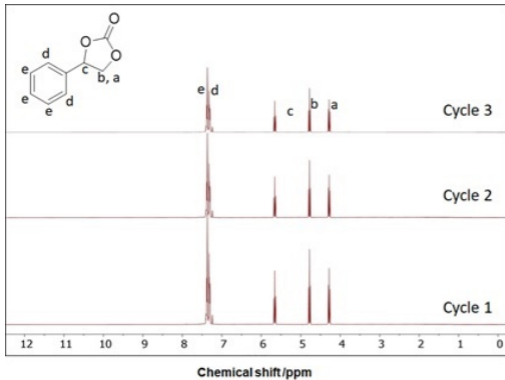

Supplement: NA-006-D4NA00535J-s005 [file NA-006-D4NA00535J-s005.pdf]

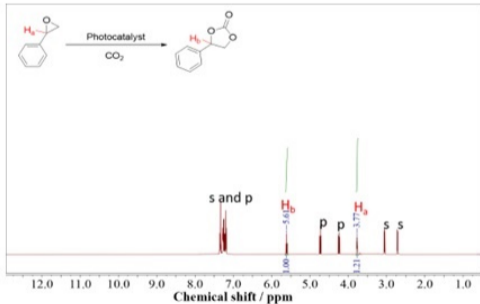

Supplement: NA-006-D4NA00535J-s006 [file NA-006-D4NA00535J-s006.pdf]

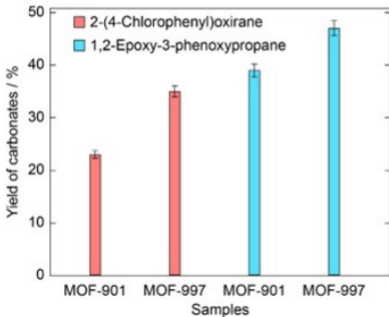

Supplement: NA-006-D4NA00535J-s007 [file NA-006-D4NA00535J-s007.pdf]
