# Supplementary material for: Titanium metal–organic frameworks for photocatalytic CO2 conversion through a cycloaddition reaction
Source: Nanoscale Adv. 2024 Aug 16;6(19):4804–13. doi: 10.1039/d4na00535j (PMC11391913; doi:10.1039/d4na00535j)
Supplement: NA-006-D4NA00535J-s002 [file NA-006-D4NA00535J-s002.pdf]

Supplementary Information (SI) for Nanoscale Advances.
This journal is © The Royal Society of Chemistry 2024

**Table S3.** Cycloaddition of $CO_2$ and styrene oxide under neat (green) conditions

| Entry | Photocatalyst[a] | Catalyst (mol%) | Yield%[b] | TON[c] | TOF ($h^{-1}$)[d] |
|---|---|---|---|---|---|
| 1 | MOF-901, *n*-$Bu_4$NBr, light | 0.005 | 99.9 | 64.4 | 2.7 |
| 2 | MOF-997, *n*-$Bu_4$NBr, light | -- | 99.9 | -- | -- |
| 3 | MOF-997-O, $^n$$Bu_4$NBr, light | -- | 99.9 | -- | -- |
| 4 | *n*-$Bu_4$NBr, no catalyst, light | -- | 23 | -- | -- |
| 5 | MOF-901 light | 0.005 | 0 | 0 | 0 |
| 6 | MOF-901, *n*-$Bu_4$NBr, no light | 0.005 | 0 | 0 | 0 |
| 7 | MOF-901, no light | 0.005 | 0 | 0 | 0 |
| 8 | *n*-$Bu_4$NBr, no catalyst, no light | -- | 0 | 0 | 0 |
| 9 | MOF-901, *n*-$Bu_4$NBr, no light, heat (353 K) | 0.005 | 25 | 16 | 0.7 |
| 10 | $TiO_2$, *n*-$Bu_4$NBr, light | -- | 50 | -- | -- |
| 11 | $TiO_2$, light | -- | 12 | -- | -- |
| 12 | No *n*-$Bu_4$NBr, no catalyst, light | -- | 0 | -- | -- |

[a]Reaction condition: styrene oxide (0.44 mmol), photocatalyst (10 mg), *n*-$Bu_4$NBr (9 mg, 0.028 mmol), and 0.045 mmol carbon dioxide at 353 K and 24 h in 4 mL of acetonitrile/methanol (3:1 v:v); [b]Conversion determined by $^1$H NMR; [c]TON: turnover number = (mmol of product)/(mmol of catalyst); [d]TOF: turnover frequency = (mmol of product)/(mmol of catalyst)(reaction time, hour); [b]Conversion determined by $^1$H-NMR.
