# Supplementary material for: Titanium metal–organic frameworks for photocatalytic CO2 conversion through a cycloaddition reaction
Source: Nanoscale Adv. 2024 Aug 16;6(19):4804–13. doi: 10.1039/d4na00535j (PMC11391913; doi:10.1039/d4na00535j)
Supplement: NA-006-D4NA00535J-s008 [file NA-006-D4NA00535J-s008.pdf]

Supplementary Information (SI) for Nanoscale Advances.
This journal is © The Royal Society of Chemistry 2024

Supporting Information

Titanium Metal–Organic Frameworks for Photocatalytic CO_2 Conversion through Cycloaddition Reaction

James Kegere,[1] Shaikha S. Alneyadi,[1] Alejandro Perez Paz,[1] Lamia A. Sidiq,[1] Afra Alblooshi,[1] Mohamed A. Alnaqbi,[1] Ahmed Alzamly[1,*], Yaser Greish,[1,2,*]

[1]Department of Chemistry, College of Science, and [2]Zayed Centre for Health Sciences, United Arab Emirates University, Al Ain 15551, UAE

[*]E-mails: y.afifi@uaeu.ac.ae (Y.G.); ahmed.alzamly@gmail.com (A.A.)

Table of Contents

**Section S1**	Materials and methods	S3
**Section S2**	Synthesis of raw materials and MOFs	S4–S5
**Section S3**	Powder X-ray diffraction	S5
**Section S4**	N_2 and CO_2 sorption	S6
**Section S5**	Thermogravimetric analysis (TGA)	S6
**Section S6**	Scanning electron microscopy (SEM)	S7
**Section S7**	UV-Vis diffuse reflectance spectroscopy	S7
**Section S8**	Cycloaddition reactions of CO_2	S7–S10
**Section S9**	References	S10

Section S1: Materials and methods

Chemicals:

All chemicals in this study were used as purchased from Sigma Aldrich without further purification: 4-aminobenzoic acid (99% purity), benzene-1,4-dialdehyde (97% purity), titanium(IV) isopropoxide (98% purity), potassium persulfate (oxone), methanol (99.9% extra dry grade), isopropanol (99% purity), *N*,*N*-dimethylacetamide (DMA, 99.9% extra grade), *N*,*N*-dimethylformamide (DMF, 99.8% extra dry grade), dichloromethane (99.8% extra dry grade), acetonitrile, potassium bromide, calcium carbonate, tetrabutylammonium hexafluorophosphate (98% purity), phenyl epoxide, deuterated chloroform ($CDCl_3$, 99.9% purity), and dimethyl sulfoxide-d_6 (DMSO-d_6, 99.9% purity).

Analytical techniques:

Powder X-ray diffraction (PXRD) was measured using Rigaku Mini-Flex benchtop X-ray diffractometer having CuKα radiation tube (λ = 1.542 Å) operated at 40 kV across a range of 3−30° 2θ and a rate of 0.5° min^{-1}. Fourier-transform infrared spectroscopy (FTIR) was collected using Agilent Technologies Cary 600 series spectrometer. Morphological features of the MOFs were analyzed using a Quattro ESEM fitted with energy dispersive X-ray spectroscopy (EDX) detector that was used to study elemental mapping of the samples. Thermogravimetric analysis (TGA) was carried out using Mettler Toledo instrument TGA-2 analyzer using N_2 airflow and aluminum pan as sample holders. Porosity was determined through N_2 sorption measurements at 77 K using a micromeritics Tri-Star 11 plus gas sorption instrument. UV-Vis diffuse reflectance spectroscopy for the bandgap determination was obtained using Shimadzu UV-3600. Photocycloaddition reactions were conducted using xenon lamp as source of visible light and the experiment was performed in sealed tubes under stirring conditions. Proton nuclear magnetic resonance (^{1}H-NMR) spectra were collected using Varian 400 MHz spectrometer with chloroform-d and dimethyl-sulfoxide-d solvents. Solid-state NMR spectra were collected using a 11.4 T magnet on a Bruker Advance I spectrometer operating at 125.75 MHz for ^{13}C-NMR.

Section S2: Synthesis of raw materials and MOFs

Synthesis of raw materials

Synthesis of imine linker[3]. The synthesis process began by combining 4-aminobenzoic acid with 1,4-benzene dialdehyde at a 2.1 mole ratio in 2 mL of methanol, followed by a 30-minute evacuation. To this mixture, 2-3 drops of pyrimidine were introduced, and the reaction was left stirring in an ice bath for 18 hours. The resulting yellow solid was washed in methanol, subjected to crystallization, and subsequently characterized using ^{1}H NMR, FT-IR, and PXRD techniques (Scheme S1).

Synthesis of amide linker[4]. Initially, 1.15 grams (8.37 mmol) of solid 4-aminobenzoic acid dissolved in 15 mL of NMP was chilled to 5°C in an ice bath. Over the course of an hour, 0.812 grams (4.00 mmol) of solid terephthaloyl chloride was cautiously added. The reaction mixture was stirred at 5°C for 2 hours and then allowed to continue stirring at room temperature for an additional 12 hours. Following this, 20 mL of distilled water was added. The resulting product underwent filtration and subsequent

washing: initially with 20 mL of NMP, then five washes with 50 mL of distilled water, two rinses with 20 mL of methanol, and finally, it was dried at 65°C for 12 hours to yield 4,4'-(terephthaloylbis(azanediyl))dibenzoic acid (Scheme S1).

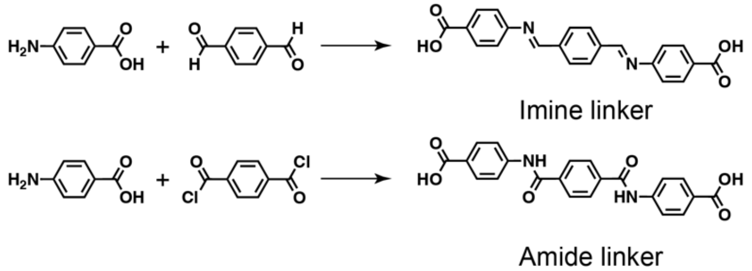

Scheme S1. Synthesis and chemical structures of imine and amide linkers that comprise MOF-901 and MOF-997, respectively.

Synthesis of Ti-oxo cluster[5]. Starting with 16 milligrams of 4-amino benzoic acid (0.116 mmol) dissolved in a 2 mL mixture of isopropanol and methanol (1:1), the solution underwent evacuation before the addition of 9 microliters of Titania isopropoxide. This mixture was then transferred into a tightly sealed Teflon-lined autoclave and subjected to an oven at 140 degrees Celsius for 72 hours (approximately 3 days). Post-reaction, the resulting yellow crystals were washed successively with methanol and dimethyl acetamide, followed by activation in dichloromethane for 24 hours. The obtained yellow solid was prepared for further characterization.

Synthesis of MOFs

MOF-901. Was synthesized following a procedure reported in the literature[1] with slight modifications. Each experiment consisted of 16 mg of 4-amino benzoic acid (0.116 mmol) dissolved in 2 mL of isopropanol:methanol (1:1 mL). A mixture of isopropanol and methanol (1:1 mL) was used to dissolve 10 mg of benzene-1,4-dialdehyde (0.074 mmol). Subsequently, 9 μL of titanium isopropoxide (0.032 mmol) was added into the 4-aminobenzoic acid solution. The two mixtures were then mixed in a Teflon lined autoclave and tightly capped. The reaction mixture was heated at 140 °C in a preheated oven for 72 hours. The yellowish brown solid, MOF-901, was filtered and washed with methanol, then DMA for 12 hours, followed by Soxhlet extraction for another 24 hours using chloroform. The samples were solvent exchanged by soaking in dichloromethane for 36 hours and the activation was executed under dynamic vacuum at 120 °C for 24 hours.

MOF-997. Was synthesized by post-synthetic oxidation of MOF-901. The oxidation was done by using oxone as the oxidant agent. Particularly, oxone (30 mg, 0.112 mmol) and 30 mg of MOF-901 were loaded into a 20-mL vial. DMF (5 mL) and glacial acetic acid (1 mL) were then added to the vial. The solution was stirred for 5 hours at room temperature. The solid was then collected through filtration, washed with sodium thiosulfate (10% aqueous solution), water, and tetrahydrofuran. The oxidized sample was solvent exchanged using Soxhlet extraction with chloroform for 24 hours. The sample was then washed with acetone, methanol, and dichloromethane before being activated under dynamic vacuum at 120 °C for 24 hours.

Section S3: Powder X-ray diffraction

The activated MOFs were loaded onto a holding disc (the sample holder) and placed in the chamber of the X-ray instrument at room temperature. The samples were measured from 3−30° 2θ with a scanning rate of 0.5° min^{-1}.

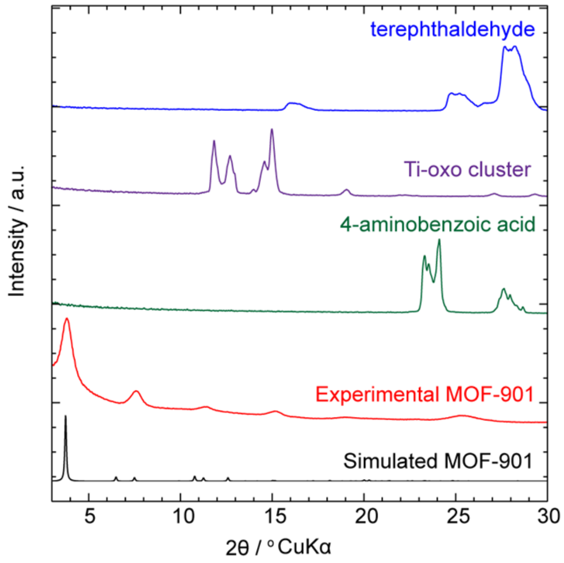

Figure S1. a) Overlay of PXRD patterns of simulated PXRD pattern of MOF-901(black), as-synthesized MOF-901(red), benzene-1,4-dialdehyde (blue) 4-aminobenzoic acid (green), and Ti-oxo cluster (purple)

Section S4: N_2 and CO_2 Sorption

The solvent-exchanged sample was loaded into a pre-weighted sorption cell. The sample was then activated at 120 °C for 24 hours and then re-weighed before being securely placed in the holding chamber of the sorption instrument. Liquid nitrogen in the flask was added to the stage to ensure the sorption cell within the chamber can be fully immersed. The experiment was initiated and allowed to run until all adsorption and desorption points were completed.

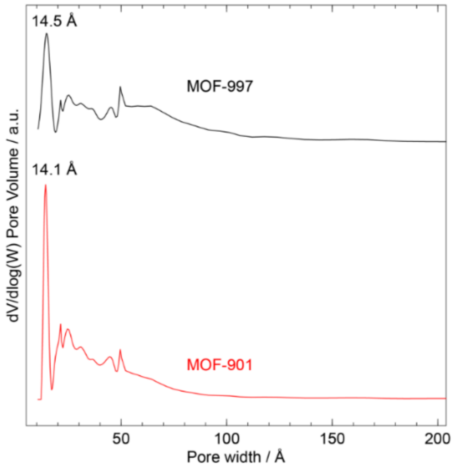

Figure S2. Pore size distribution for MOF-901 and MOF-997, estimated using DFT calculation (cylinder geometry

and NLDFT for pillared clay model), reveals a prominent micropore peak at 14.1 and 14.5 Å, respectively. The broader peaks at 22 Å may be attributed to the pores resulting from interparticle spaces within the MOFs.

Section S5: Thermogravimetric analysis (TGA)

Samples, weighing up to 5 mg each, were positioned on alumina sample holder pans. The samples were then heated to 800 °C with a heating rate of 5 °C per minute.

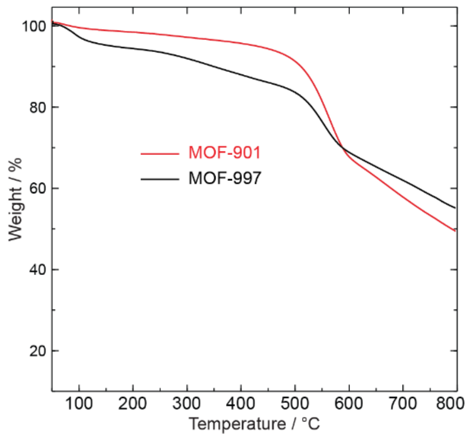

Figure S3. TGA profiles of MOF-901 and MOF-997 measured under nitrogen flow.

Section S6: Scanning electron microscopy (SEM)

Samples, each measuring 1 cm^2, were affixed to FESEM stubs using carbon tape and rendered conductive by applying a thin platinum coating with a sputter coater. These prepared samples were subsequently placed in the SEM's sample holding chamber, where various parameters were adjusted for the morphological analysis. Additionally, EDX analysis was conducted using the same instrument to determine the elemental composition percentages in the analyzed samples.

Table S1. EDXS-Atomic percentages of different elements in MOF-901 and MOF-997

**Element**	**MOF-901**	**MOF-997**
Carbon	52.93	33.84
Titanium	7.18	13.90
Oxygen	39.9	52.27

Section S7: UV-Vis diffuse reflectance spectroscopy

To determine the bandgap of MOF-901 and MOF-997, the samples were pressed onto calcium carbonate and loaded into the instrument's sample holding chamber. After adjusting the parameters, a UV-Vis beam was directed at the sample. The data obtained was then used to create plots of absorbance vs. wavelength, and bandgap estimates were calculated using Tauc plots.

Section S8: Cycloaddition reactions of CO_2

Cycloaddition reactions of CO_2 were carried out through a procedure reported in the literature.[2] Different sample weights of MOF-901 and MOF-997 (5 mg, 10 mg, 15 mg, and 20 mg) were prepared and

placed in sealed tubes. Each tube was loaded with 9 mg of co-catalyst tetrabutylammonium bromide (TBAB). Subsequently, 54 μL of styrene-oxide was added to each reaction tube, along with a magnetic bar. Finally, 3 mg of solid CO_2 was added to each reaction tube, and the tubes were promptly sealed to prevent CO_2 leakage. The reaction samples were transferred to a halogen lamp (380–780 nm wavelength range) with no cut-off filter positioned adjacent to the magnetic stirrers, where the samples were securely placed. The reaction proceeded for 24 hours. The products were obtained by washing the samples with acetonitrile and filtering them using nylon syringe filters. The collected colorless product was dried using a rotavapor at room temperature, operating at 400 rpm for 30 minutes. The dried solid sample was subsequently dissolved in deuterated chloroform for ^{1}H-NMR analysis.

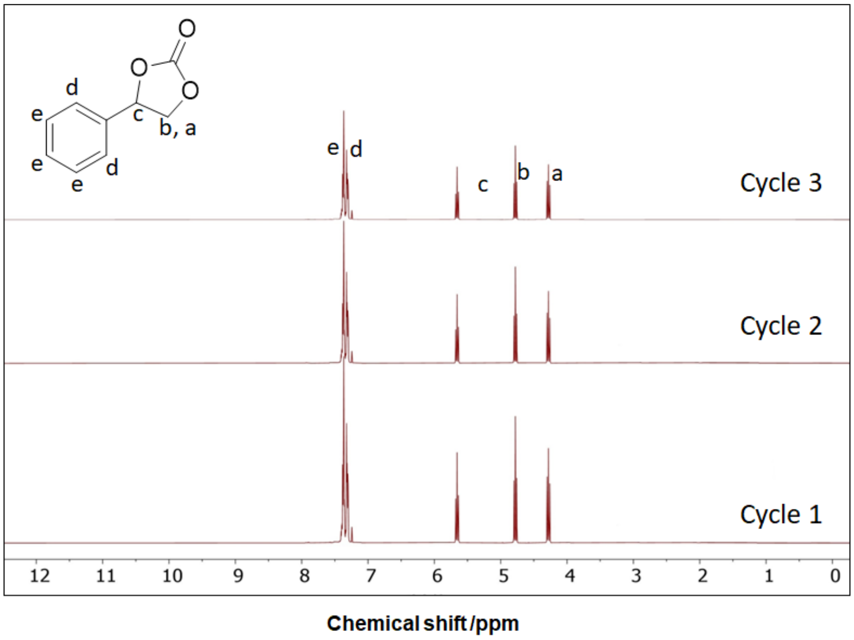

Figure S4. ^{1}H-NMR spectra of the styrene carbonate product catalyzed by MOF-997 for three consecutive cycles.

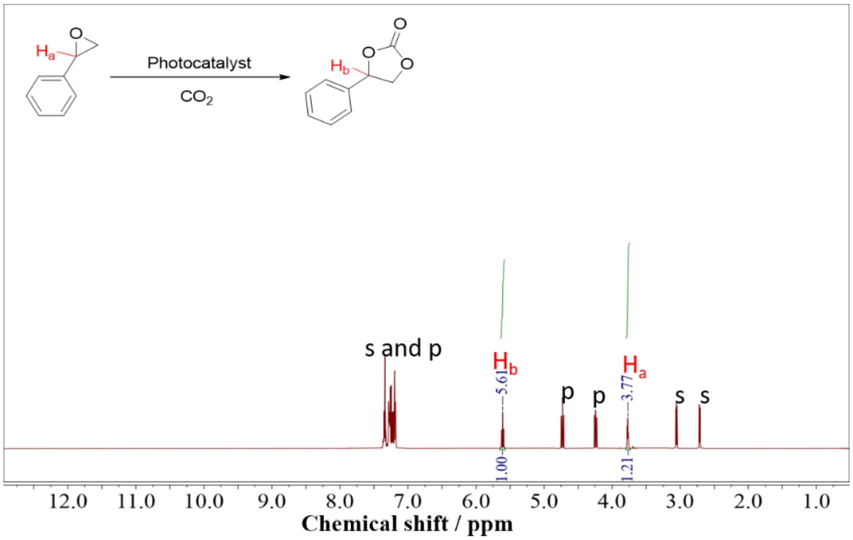

Figure S5. ^{1}H-NMR of spectra of the styrene carbonate product catalyzed by the amide linker.

Table S2. Styrene carbonate conversion catalyzed by imine, amide linkers, and Ti-oxo cluster

**Material**	**Conversion (%)**
Imine linker	16.0
Amide linker	45.0
Ti-oxo cluster	33.0

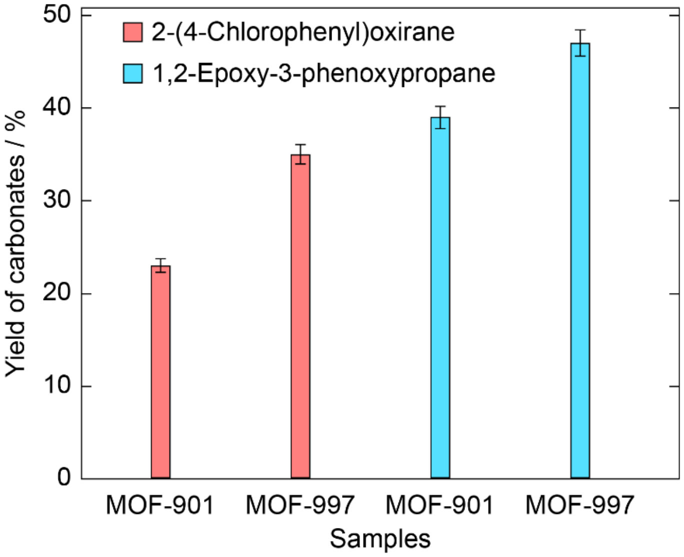

Figure S6. Percentage yield of cycloaddition of CO_2 to 2-(4-chlorophenyl) oxirane and 1,2-epoxy-3-phenoxypropane to the corresponding carbonates photocatalyzed by MOF-901 and MOF-997.

Table S3. Cycloaddition of CO_2 and styrene oxide under neat (green) conditions

Entry	Photocatalyst[a]	Catalyst (mol%)	Yield%[b]	TON[c]	TOF (h^{-1})[d]
1	MOF-901, *n*-Bu_4NBr, light	0.005	99.9	64.4	2.7
2	MOF-997, *n*-Bu_4NBr, light	--	99.9	--	--
3	MOF-997-O, $^nBu_4NBr$, light	--	99.9	--	--
4	*n*-Bu_4NBr, no catalyst, light	--	23	--	--
5	MOF-901 light	0.005	0	0	0
6	MOF-901, *n*-Bu_4NBr, no light	0.005	0	0	0
7	MOF-901, no light	0.005	0	0	0
8	*n*-Bu_4NBr, no catalyst, no light	--	0	0	0
9	MOF-901, *n*-Bu_4NBr, no light, heat (353 K)	0.005	25	16	0.7
10	TiO_2, *n*-Bu_4NBr, light	--	50	--	--
11	TiO_2, light	--	12	--	--
12	No *n*-Bu_4NBr, no catalyst, light	--	0	--	--

[a]Reaction condition: styrene oxide (0.44 mmol), photocatalyst (10 mg), *n*-Bu_4NBr (9 mg, 0.028 mmol), and 0.045 mmol carbon dioxide at 353 K and 24 h in 4 mL of acetonitrile/methanol (3:1 v:v); [b]Conversion determined by ^1H NMR; [c]TON: turnover number = (mmol of product)/(mmol of catalyst); [d]TOF: turnover frequency = (mmol of product)/(mmol of catalyst)(reaction time, hour); [b]Conversion

determined by ^{1}H-NMR.

Section 9: References

[1] H. L. Nguyen, F. Gándara, H. Furukawa, T. L. H. Doan, K. E. Cordova, O. M. Yaghi, *J. Am. Chem. Soc.* **2016**, *138*, 4330–4333.
[2] L. A. Siddig, R. H. Alzard, H. L. Nguyen, C. R. Göb, M. A. Alnaqbi, A. Alzamly, *ACS Omega* **2022**, *7*, 9958–9963.
[3] Z. Zhang, Q. Wang, D. Xue, J. Bai, *Chem. Asian J.* **2019**, *14*, 3603–3610.
[4] A. Sima, B. Ghodsieh, F. Alireza, *Inorg. Chem. Commun.* **2017**, *81*, 37–42.
[5] H. Keunil, C. Hyungphil, *Inorg. Chem.* **2013**, *52*, 970–9707.
