# Supplementary material for: Titanium metal–organic frameworks for photocatalytic CO2 conversion through a cycloaddition reaction
Source: Nanoscale Adv. 2024 Aug 16;6(19):4804–13. doi: 10.1039/d4na00535j (PMC11391913; doi:10.1039/d4na00535j)
Supplement: NA-006-D4NA00535J-s009 [file NA-006-D4NA00535J-s009.pdf]

Supplementary Information (SI) for Nanoscale Advances.
This journal is © The Royal Society of Chemistry 2024

Table S1. EDXS-Atomic percentages of different elements in MOF-901 and MOF-997

**Element**	**MOF-901**	**MOF-997**
Carbon	52.93	33.84
Titanium	7.18	13.90
Oxygen	39.9	52.27
