# Supplementary material for: Titanium metal–organic frameworks for photocatalytic CO2 conversion through a cycloaddition reaction
Source: Nanoscale Adv. 2024 Aug 16;6(19):4804–13. doi: 10.1039/d4na00535j (PMC11391913; doi:10.1039/d4na00535j)
Supplement: NA-006-D4NA00535J-s010 [file NA-006-D4NA00535J-s010.pdf]

Supplementary Information (SI) for Nanoscale Advances.
This journal is © The Royal Society of Chemistry 2024

Table S2. Styrene carbonate conversion catalyzed by imine, amide linkers, and Ti-oxo cluster

**Material**	**Conversion (%)**
Imine linker	16.0
Amide linker	45.0
Ti-oxo cluster	33.0
